# Industrial Trans Fatty Acids Stimulate SREBP2‐Mediated Cholesterogenesis and Promote Non‐Alcoholic Fatty Liver Disease

**DOI:** 10.1002/mnfr.201900385

**Published:** 2019-08-07

**Authors:** Antwi‐Boasiako Oteng, Anke Loregger, Michel van Weeghel, Noam Zelcer, Sander Kersten

**Affiliations:** ^1^ Nutrition, Metabolism and Genomics Group Division of Human Nutrition and Health Wageningen University 6708 WE Wageningen The Netherlands; ^2^ Department of Medical Biochemistry Academic Medical Center University of Amsterdam 1105 AZ Amsterdam The Netherlands; ^3^ Laboratory Genetic Metabolic Diseases Amsterdam UMC, University of Amsterdam, Amsterdam Gastroenterology and Metabolism, Amsterdam Cardiovascular Sciences 1105 AZ Amsterdam The Netherlands

**Keywords:** cholesterogenesis, cholesterol metabolism, elaidate, non‐alcoholic fatty liver disease, sterol regulatory element binding proteins

## Abstract

**Scope:**

The mechanisms underlying the deleterious effects of trans fatty acids on plasma cholesterol and non‐alcoholic fatty liver disease (NAFLD) are unclear. Here, the aim is to investigate the molecular mechanisms of action of industrial trans fatty acids.

**Methods and results:**

Hepa1‐6 hepatoma cells were incubated with elaidate, oleate, or palmitate. C57Bl/6 mice were fed diets rich in trans‐unsaturated, cis‐unsaturated, or saturated fatty acids. Transcriptomics analysis of Hepa1‐6 cells shows that elaidate but not oleate or palmitate induces expression of genes involved in cholesterol biosynthesis. Induction of cholesterogenesis by elaidate is mediated by increased sterol regulatory element‐binding protein 2 (SREBP2) activity and is dependent on SREBP cleavage–activating protein (SCAP), yet independent of liver‐X receptor and ubiquitin regulatory X domain‐containing protein 8. Elaidate decreases intracellular free cholesterol levels and represses the anticholesterogenic effect of exogenous cholesterol. In mice, the trans‐unsaturated diet increases the ratio of liver to gonadal fat mass, steatosis, hepatic cholesterol levels, alanine aminotransferase activity, and fibrosis markers, suggesting enhanced NAFLD, compared to the cis‐unsaturated and saturated diets.

**Conclusion:**

Elaidate induces cholesterogenesis in vitro by activating the SCAP–SREBP2 axis, likely by lowering intracellular free cholesterol and attenuating cholesterol‐dependent repression of SCAP. This pathway potentially underlies the increase in liver cholesterol and NAFLD by industrial trans fatty acids.

## Graphical abstract

**The cholesterogenic effect of the trans fatty acid elaidate** is due in part to its ability to reduce levels of intracellular cholesterol, and reduce the sensitivity of sterol regulatory element binding protein (SREBP) cleavage–activating protein (SCAP) to cholesterol. These properties result in the activation of the SREBP signaling pathway, leading to increased expression of genes involved in cholesterol biosynthesis.

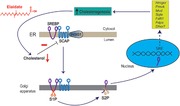

## 1 Introduction

Trans fatty acids are unsaturated fatty acids with at least one double bond in the trans configuration. Compared to the more common cis‐unsaturated fatty acids, trans‐unsaturated fatty acids are less flexible and maintain a straight chain carbon backbone, similar to saturated fatty acids.^1^, ^2^ Part of the trans fatty acids consumed by humans are industrially produced by partial hydrogenation of vegetable oils and are present in deep fried foods, pastries, cookies, margarine, and popcorns. The other set of trans fatty acids are synthesized naturally in the gut of ruminants by bacterial biohydrogenation and are present in meat and dairy products of cattle, goat, and sheep.^3^, ^4^, ^5^ Partially hydrogenated vegetable oils and trans fatty acids are used by the food industry because they confer a combination of beneficial properties to food products, including a long shelf life and a pleasant mouthfeel. In response to growing evidence linking intake of trans fatty acids to coronary heart disease, most food manufacturers and retailers have largely removed trans fatty acids from their products. The 2018 Dutch Nutrition Survey indicated that nowadays, trans fatty acids only provide ≈0.3% of the daily energy requirement, as opposed to 5–10% several decades ago.

In a landmark study in 1990, Mensink and Katan demonstrated the plasma cholesterol–raising effect of trans fatty acids in human volunteers.^6^ Specifically, they found that trans fatty acids raise plasma low density lipoprotein (LDL) cholesterol and reduce plasma high density lipoprotein (HDL) cholesterol levels. Following this important finding, multiple epidemiological studies found strong positive associations between consumption of industrial trans fatty acids and the onset and progression of coronary heart disease.^7^, ^8^, ^9^, ^10^ A recent meta‐analysis convincingly established that intake of trans fatty acids is positively associated with all‐cause mortality, total coronary heart disease, and coronary heart disease mortality.^11^

In addition to coronary heart disease, trans fatty acids have also been connected with other diseases, including non‐alcoholic fatty liver disease.^12^, ^13^, ^14^ Indeed, several studies have shown that feeding mice a diet rich in industrial trans fatty acids enhances liver steatosis and features of NASH.^15^, ^16^, ^17^, ^18^, ^19^ The increase in liver triglycerides was associated with elevated expression of lipogenic genes, such as *Fasn*, *Acaca*, and *Srebp1*. Remarkably, little is known about the mechanism(s) that underlie the increase in liver triglycerides and lipogenic gene expression in mice fed diets rich in trans fatty acids.

Because the health concerns connected to the consumption of industrial trans fatty acids have been largely allayed by the removal of trans fatty acids from foods, unfortunately there has been little effort into trying to understand the molecular mechanisms underlying the detrimental effects of trans fatty acids. In a recent study, we found that the industrial trans fatty acid elaidate and the saturated fatty acid palmitate had very distinct effects on markers of inflammation and the unfolded protein response in murine RAW264.7 macrophages. In addition, it was observed that elaidate upregulated the expression of putative target genes of sterol regulatory element binding proteins (SREBPs),^20^ an observation that was further confirmed in studies in human hepatoma HepG2 and Huh7 cells.^21^, ^22^

SREBPs, consisting of SREBP1 and SREBP2, are basic helix‐loop‐helix–leucine zipper (bHLH‐Zip) transcription factors involved in the regulation of cellular lipid metabolism.^23^, ^24^, ^25^ SREBP1 preferentially induces genes involved in fatty acid synthesis, while SREBP2 is more selective for genes involved in cholesterol biosynthesis.^26^, ^27^ SREBPs are synthesized as inactive precursors and held in a tripartite complex with SREBP cleavage–activating protein (SCAP) and INSIGs in the endoplasmic reticulum (ER) membrane. In response to low cholesterol levels, INSIG dissociates and the SCAP–SREBP complex is translocated to the Golgi apparatus for proteolytic activation. The resulting active *N*‐terminal SREBP enters the nucleus, where it binds to sterol regulatory elements in the promoter regions of target genes.^27^, ^28^, ^29^, ^30^, ^31^ Although both cholesterol and fatty acids are products of SREBP signaling, regulation of SREBP by fatty acids is relatively poorly delineated compared to regulation by cholesterol. Evidence has been presented that poly‐unsaturated fatty acids decrease *Srebp1* mRNA levels,^32^, ^33^, ^34^ stimulate SREBP1 decay,^35^ inhibit proteolytic processing of SREBP1 via binding to the auxiliary protein ubiquitin regulatory X domain‐containing protein 8 (UBXD8),^36^, ^37^ and antagonize the activity of the liver X receptor (LXR), which is a potent inducer of *Srebp1* gene transcription.^38^, ^39^ How elaidate causes activation of the SREBP pathway remains unclear.

Here, we set out to better understand the mechanism of action of industrialized trans fatty acids. To that end, we pursued two independent experimental strategies. On the one hand, we conducted in vitro experiments in hepatocyte and adipocyte cell lines treated with individual fatty acids, and on the other hand, we fed mice diets enriched in trans‐unsaturated fatty acids, cis‐unsaturated fatty acids, and saturated fatty acids, and analyzed the hepatic phenotype.

## 2 Experimental Section

### 2.1 Animal Treatment

Animal studies were performed using 4‐ to 6‐month‐old pure‐bred male mice on a C57Bl/6 background. For the long‐term experiment, the mice were randomly assigned to three groups (*n* = 8 mice per group). For 7 weeks, each group was fed a high fat diet rich in trans‐unsaturated (Trans), cis‐unsaturated (Cis), or saturated (Saturated) fatty acids (modified TestDiet 58V8; TestDiet LTD, London). A detailed composition of the test diets is provided in Table S1, Supporting Information. Fat sources were from partially hydrogenated soy oil for the Trans diet, canola and palm olein oil for the Cis diet, and cocoa butter for the Saturated diet. Total fat content in all three diets was 45 energy percent. Bodyweight and food intake were measured weekly. Following the diet intervention, mice were anesthetized with isoflurane, blood was collected via orbital puncture, and mice were euthanized by cervical dislocation. Tissues for RNA and protein analysis were immediately frozen in liquid nitrogen and stored at −80 °C. Tissues for histological analysis were fixed in 4% paraformaldehyde and later embedded in paraffin. For the short‐term experiment, the mice were randomly assigned to three diet groups (*n* = 10 mice per group). The mice were fasted from 12:00 to 18:00 h, after which they were given access to one of the three diets. The mice were euthanized the next day between 9:00 and 11:00 h as described above. The animal studies were approved by the Local Animal Ethics Committee at Wageningen University (2014096.d and AVD104002015236: 2016.W‐0093.006).

### 2.2 FA Profiling of Test Diets by Gas Chromatography‐Flame Ionization Detection

The Folch technique was used to extract the fat component in the test diets as previously described.^40^ In summary, a 2:1 mixture of chloroform to ethanol was used to isolate fats from diets. Water was then added to obtain a biphasic system, with the fat present in the lower chloroform phase. The extracted fats were measured gravimetrically after purification followed by saponification with methanolic NaOH and methylation with boron trifluoride to obtain fatty acid methyl esters. The methyl esters were then fractionated by gas chromatography, detected by flame ionization, and calculated as a fraction of the total amount.

### 2.3 Measurement of Plasma Parameters

Blood samples collected into EDTA‐coated tubes were centrifuged at 10 000 g for 15 min at 4 °C to obtain plasma. Plasma levels of non‐esterified fatty acids (NEFA) and glycerol were measured using kits from HUMAN Diagnostics (Wiesbaden, Germany) according to manufacturer's protocol. Plasma cholesterol and glucose were also quantified using kits from Diasys Diagnostics Systems (Holzheim, Germany) according to manufacturer's protocol. Plasma serum amyloid A (SAA) (Tridelta Development Ltd., Ireland) and haptoglobin (Abcam, Cambridge) were measured using ELISA kits with slight modifications to the manufacturer's protocol. Plasma alanine aminotransferase (ALT) activity (Abcam, Cambridge, UK) and insulin (Crystal Chem, Downers Grove, IL) were also measured using dedicated kits.

### 2.4 Liver Hematoxylin and Eosin Staining

Histology of mice liver samples was performed by hematoxylin and eosin (H&E) staining. In summary, paraformaldehyde‐fixed liver tissues were processed using ethanol and xylene before being embedded into paraffin blocks. Thin liver sections at 5 µm thickness were made using a microtome onto superfrost glass slides and incubated at 37 °C overnight. The liver slices were then stained for 10 min in Mayer hematoxylin solution and for 10 s in eosin Y solution. The slides were then observed under a light microscope and representative images taken.

### 2.5 Liver Collagen Staining

Collagen staining of the liver was performed using the Fast Green FCF/Sirius Red staining. In brief, frozen livers were sectioned at 5 µm, mounted on glass slides and left to air dry for 30 min at room temperature. The slides were then fixed in 4% paraformaldehyde for 30 min, washed thrice (1 min each) in absolute ethanol and rinsed briefly with demi water. The tissues were then stained in collagen dye (0.1% Fast Green FCF, 0.1% direct Red 80 in saturated picric acid solution, Sigma) in a 37 °C incubator for 90 min. The tissues slices were then rinsed with demi water for three to four times until the water was clear, dehydrated with absolute ethanol thrice (30 s each) and in xylene twice (5 min each). The slides were air dried and mounted with Depax mounting medium. Pictures were taken by light microscope.

### 2.6 Liver Triglyceride Measurement

To measure liver triglycerides, 2% liver homogenates were made in a buffer composed of 10 mm Tris, 2 mm EDTA, and 25 mm sucrose at pH 7.5 by homogenizing in a TissueLyser II (Qiagen, Hilden, Germany). Liver triglyceride content was then quantified using Triglyceride liquicolor^mono^ from HUMAN Diagnostics (Wiesbaden, Germany) according to manufacturer's instructions.

### 2.7 Liver Cholesterol Measurement

Total cholesterol in liver was quantified by gas chromatography‐mass spectroscopy (GC‐MS) as previously described.^41^ In brief, a procedure involving extraction, centrifugation, and purification steps were used to isolate cholesterol from 200 mg liver samples using KOH, water, and hexane as solvents. The extracts along with calibration standards were dissolved in dimethyl formamide (DMF), which also served as mobile phase prior to injection into the GC. The chromatograms that were generated were analyzed as total cholesterol content in the mice liver samples.

### 2.8 RNA Isolation and Quantitative Real‐Time PCR

Total RNA was isolated from mice tissues, Hepa1‐6, Huh7, or 3T3‐L1 cells using TRIzol reagent (Invitrogen, Bleiswijk, The Netherlands) and in the case of the mice tissues, homogenized in TRIzol using a TissueLyser II (Qiagen, Venlo, The Netherlands). Following RNA isolation, 1000 ng of RNA was used to synthesize cDNA by reverse transcription using first strand cDNA synthesis kit (Thermo Fisher Scientific, Schaumburg, IL) in accordance with manufacturer's protocol. Changes in gene expression were then determined using a quantitative real‐time PCR detection system (Bio‐Rad) by using SensiMix (Bioline, GC Biotech, Alphen aan den Rijn, The Netherlands). Gene expression values were normalized to the 36b4 housekeeping gene. Primer sequences for measured genes are listed in Table S2, Supporting Information.

### 2.9 Cell Culture, 3T3‐L1 Differentiation, and siRNA Knockdown

#### 2.9.1 Cell Culture

Hepa1‐6 (# CRL‐1830) and 3T3‐L1 pre‐adipocytes (# CL‐173) were obtained from ATCC (Manassas, VA, USA). Huh‐7 cells was a gift from Nicole de Wit (Wageningen University and Research). The cells were cultured in Dulbecco's modified Eagle medium (DMEM) media supplemented with 10% fetal bovine serum, 100 U mL^−1^ penicillin and 1000 µg mL^−1^ streptomycin (Lonza, Verviers, Belgium) in a humidified chamber at 37 °C with 5% CO_2_.

#### 2.9.2 3T3‐L1 Differentiation

During differentiation, the pre‐adipocytes were cultured in 6‐well plates. Two days post‐confluence, the cell culture media was changed to induction media (0.5 mm IBMX, 5 µg mL^−1^ insulin, 1 µm dexamethasone) for 2 days. Induction media was then replaced with insulin media (5 µg mL^−1^ insulin) for 3 days, after which the fully differentiated cells were treated with fatty acids.

#### 2.9.3 3T3‐L1 Co‐Differentiation with Fatty Acids

Pre‐adipocytes were differentiated in the presence of elaidate, oleate, or palmitate to assess the effect of trans, cis, and saturated fatty acids, respectively, on adipocyte differentiation. The 3T3‐L1 pre‐adipocytes were cultured in 6‐well plates. Two days post‐confluence, the cell culture media was changed to induction media (0.5 mm IBMX, 5 µg mL^−1^ insulin, 1 µm dexamethasone) supplemented with 500 µm fatty acids for 2 days. Induction media was then replaced with insulin media (5 µg mL^−1^ insulin) also supplemented with 500 µm fatty acids for 3 days after which the cells were harvested for gene expression analysis.

#### 2.9.4 siRNA‐Mediated Silencing

Silencing of *Srebp1*, *Srebp2*, *Scap, Ubxd8*, and *Soat1* was performed using Dharmacon ON‐TARGETplus SMARTpool siRNAs diluted in 1× siRNA buffer. Hepa1‐6 cells were plated at 5 × 10^4^ cells per well into 24‐well plates and transfected overnight with 50 nm siRNA using 2 µL per well Lipofectamine RNAiMAX transfection reagent (Life Technologies, Bleiswijk, The Netherlands). Transfected cells were subsequently treated with 500 µm fatty acids for 24 h.

#### 2.9.5 Placental Alkaline Phosphatase‐Reporter Assay

The placental alkaline phosphatase (PLAP) assay was performed to measure the processing of SREBP2 in the presence of fatty acids or controls as previously described.^42^, ^43^ HEK293 cells were plated at 2 × 10^5^ cells per milliliter into 12‐well plates and incubated overnight. The cells were then co‐transfected overnight with 150 ng each of PLAP‐conjugated SREBP2 and SCAP plasmids; pCMVpCMV‐SREBP2‐PLAP and pcDNA4‐hSCAP‐MycHis, respectively, in PEI transfection medium.^42^, ^43^ The transfected cells were washed with PBS and treated for 24 h with 500 µm of fatty acids: oleate, palmitate, elaidate, or vehicle control. Treatments with 50 µg mL^−1^ of β‐MCD cholesterol (Sigma‐Aldrich) and a combination of simvastatin (Calbiochem) and mevalonic acid (Calbiochem) at 5 µg mL^−1^ each served as negative and positive controls, respectively. The PLAP assay was then performed by incubating cultured media with PNPP substrate and colorimetrically measuring the absorbance at 405 nm after 60 min as previously described.^42^

#### 2.9.6 Sub‐Cellular Localization in CHO SCAP‐GFP Cells

CHO SCAP‐GFP cells were a kind gift of Prof. Espenshade and were described previously.^44^ CHO SCAP‐GFP cells were cultured at 37 °C in an atmosphere of 8–9% CO_2_, a 1:1 mixture of Ham's F‐12 medium, and DMEM supplemented with 5% v/v FCS. Briefly, cells were generated by transfection of SCAP‐deficient SRD‐13A cells with pGFP‐SCAP, followed by selection in a 1:1 mixture of Ham's F‐12 medium and DMEM supplemented with 5% newborn calf lipoprotein‐deficient serum. pGFP‐SCAP rescued the cholesterol auxotrophy of SRD‐13A cells. CHO SCAP‐GFP cells were incubated for 24 h with 500 µm elaidate or control. The cells were analyzed by microscopy for subcellular localization of SCAP.

### 2.10 Fatty Acid Preparation and Treatment

All fatty acid stocks were initially reconstituted in absolute ethanol. Sub‐stocks of fatty acids at 25 mm were prepared in filter‐sterilized KOH at 70 mm. Complete DMEM supplemented with fatty acid–free BSA (Sigma‐Aldrich, St. Louis, MO) were filter sterilized, and incubated for 30 min with fatty acids to complex BSA to fatty acids. Ratio of BSA to fatty acids were approximately 1:2. Vehicle control treatments only contained equivalent mixture of ethanol and KOH. During treatment, Hepa1‐6, Huh7, or 3T3‐L1 cells were seeded at 2 × 10^5^ cells per milliliter and incubated overnight, followed by treatment with 500 µm concentration of fatty acids for 24 h. Treatment with any other fatty acids were performed similarly. Differentiated 3T3‐L1 cells were incubated with 1 mm final concentrations of fatty acids.

### 2.11 Elaidate Treatment in the Presence of LXR Agonist or Exogenous Cholesterol

To investigate the role of LXR, elaidate‐treated Hepa1‐6 cells were co‐incubated for 24 h with 1 µm of GW3965 (Sigma), an LXR agonist. To investigate the effect of elaidate in the presence of elevated cholesterol, Hepa1‐6 cells were co‐incubated with elaidate in the presence of 500 µm of water‐soluble cholesterol (Sigma).

### 2.12 Intracellular Cholesterol and Cholesterol Ester Quantification by HPLC

For intracellular cholesterol and cholesterol ester measurement, 4 × 10^5^ cells per well were plated into 6‐well plates. After overnight incubation, cells were treated with 500 µm of elaidate or control for 24 h. The cells were then washed three times with ice‐cold PBS and scraped into cold methanol. Cholesterol and cholesterol esters were extracted using a single‐phase extraction. A defined amount of internal standards consisted of 0.5 nmol of cholesterol ester (CE [14:0]) and 1 nmol cholesterol‐D7. The mixture was sonicated in a sonication bath for 15 min and centrifuged for 5 min at 16 000 × *g* at 4 °C. The supernatant was transferred to a glass vial and evaporated. The residue was dissolved in 150 µL chloroform/methanol (1:1 v/v). The analysis of cholesterol and cholesterol esters was performed using an HPLC‐MS system consisting of an Ultimate 3000 binary HPLC pump, a vacuum degasser, a column temperature controller, an auto sampler and a Q‐exactive plus mass spectrometer (Thermo Scientific, Waltham, MA, USA). For the cholesterol esters, 5 µL of the lipid extract was injected onto a “normal phase column” LiChrospher 2 × 250‐mm silica‐60 column, 5 µm particle diameter (Merck, Darmstadt, Germany) as previously described.^45^ For the quantification of cholesterol, 5 µL of the same lipid extracted was injected onto a “reverse phase column” Acquity UPLC HSS T3, 1.8 µm particle diameter (Waters, Milford Massachusetts, USA) and measured as previously described (https://doi.org/10.1016/j.chroma.2014.08.088).

### 2.13 Intracellular Triglyceride Quantification

After 24 h fatty acid treatment, Hepa1‐6 cells were washed twice with PBS. In 6‐well plates, 500 µL Tris‐EDTA buffer (25 mm Tris, 1 mm EDTA [pH 7.5]) was added to the cells and to prepared triglyceride standards (Instruchemie, The Netherlands). The plates were placed in −80 °C for 1 h. The plates were allowed to thaw to room temperature, followed by the addition of 200 µL of tertiary butanol and 50 µL of methanol. The plates were put to shake at 270 rpm for 15 min followed by evaporation on a hot plate at 60 °C. Monocolor reagent (Instruchemie) was then added to the wells, followed by shaking for 5 min at 250 rpm on a plate shaker. Samples and standards were transferred to 96‐well plates and absorbance was measured at 492 nm. Triglyceride content was normalized to protein content, which was quantified by the Pierce BCA protein assay (Thermo Scientific) according to manufacturer's protocol.

### 2.14 Cell cytotoxicity by Lactate Dehydrogenase Assay

Cell cytotoxicity by lactate dehydrogenase (LDH) was performed in culture media from Hepa1‐6 cells in 12‐well plates that were incubated with 500 µm of fatty acids for 24 h. As positive control, the cells were incubated with 10 µL of 10% triton X‐100 for 15 min. LDH assay was performed according to manufacturer's protocol (Roche, USA; #11644793001). In brief, 50 µL of cell‐free culture media were transferred into 96‐well plates and incubated for 10 min at room temperature with 50 µL of a mixture of LDH reagents 1 and 2 (ratio 45:1). To stop the reaction, 25 µL of 1 m HCl was added to each well, followed by absorbance reading at 490 nm.

### 2.15 Western Immunoblotting

Protein expression was determined by western immunoblotting following siRNA knockdown of specific genes and elaidate treatment in Hepa1‐6 cells using standard protocols. In summary, proteins were isolated with RIPA lysis buffer containing protease inhibitors and quantified by Pierce BCA protein assay (Thermo Scientific, USA) according to manufacturer's protocol. Proteins were prepared and 20 µg were loaded onto SDS‐PAGE gels, transferred onto PVDF membranes, and probed for specific proteins. Primary antibodies for SREBP1 (Millipore, #MABS1987), SREBP2 (Millipore, #MABS1988), acyl‐coA synthetase short chain family member 2 (ACSS2) (Cell Signaling; #3658S), 3‐hydroxy‐3‐methylglutaryl‐CoA reductase (HMGCR) (ATCC CRL‐1811, IgG‐A9, undiluted hybridoma supernatant), low density lipoprotein receptor (LDLR) (Biovision, #3839‐100), and Actin (Merck, MAB1501) were diluted in 5% milk in TBS‐T. Secondary antibodies anti‐rabbit IgG‐HRP were diluted at 1:5000 in 5% milk.

### 2.16 Microarray Analysis

Microarray analysis was performed on liver samples from the mice fed the three different diets as well as on Hepa1‐6 hepatoma and differentiated 3T3‐L1 adipocyte cells incubated with different fatty acids. RNA was purified with RNeasy Minikit columns (Qiagen) and analyzed for quality with RNA 6000 Nano chips on the Agilent 2100 bioanalyzer (Agilent Technologies, Amsterdam, The Netherlands). One microgram of RNA was used for cDNA synthesis using the First Strand cDNA synthesis kit (Thermo Scientific). Purified RNA (100 ng) was labeled with the Ambion WT expression kit (Invitrogen) and hybridized to an Affymetrix Mouse Gene 1.1 ST array plate (mouse liver, Hepa1‐6 cells) or 2.1 ST array plate (3T3‐L1 adipocytes) (Affymetrix, Santa Clara, CA). Hybridization, washing, and scanning were carried out on an Affymetrix GeneTitan platform. Scans of the Affymetrix arrays were processed using packages from the Bioconductor project. Arrays were normalized using the robust multi‐array average method.^46^, ^47^ Probe sets were defined by assigning probes to unique gene identifiers, for example, Entrez ID.^48^ For the Hepa1‐6 and 3T3‐L1 cells, the total gene set (24 973 probe sets) was filtered to only include genes with mean signal >20, yielding 10 379 and 11 504 genes, respectively. Microarray data were submitted to the Gene Expression Omnibus (accession number pending).

### 2.17 Statistical Analysis

Results were presented as mean ± SEM for animal experiments and mean ± SD for in vitro experiments. Statistical analyses were performed using Student's paired *t*‐tests or by one‐way ANOVA followed by a Turkey's post hoc multiple comparison test (GraphPad Software, Inc., La Jolla, USA). *p* < 0.05 was considered statistically significant.

## 3 Results

### 3.1 Elaidate Induces a Cholesterogenic Gene Expression Profile in Hepa1‐6 Hepatocytes and 3T3‐L1 Adipocytes

**Figure 1:**
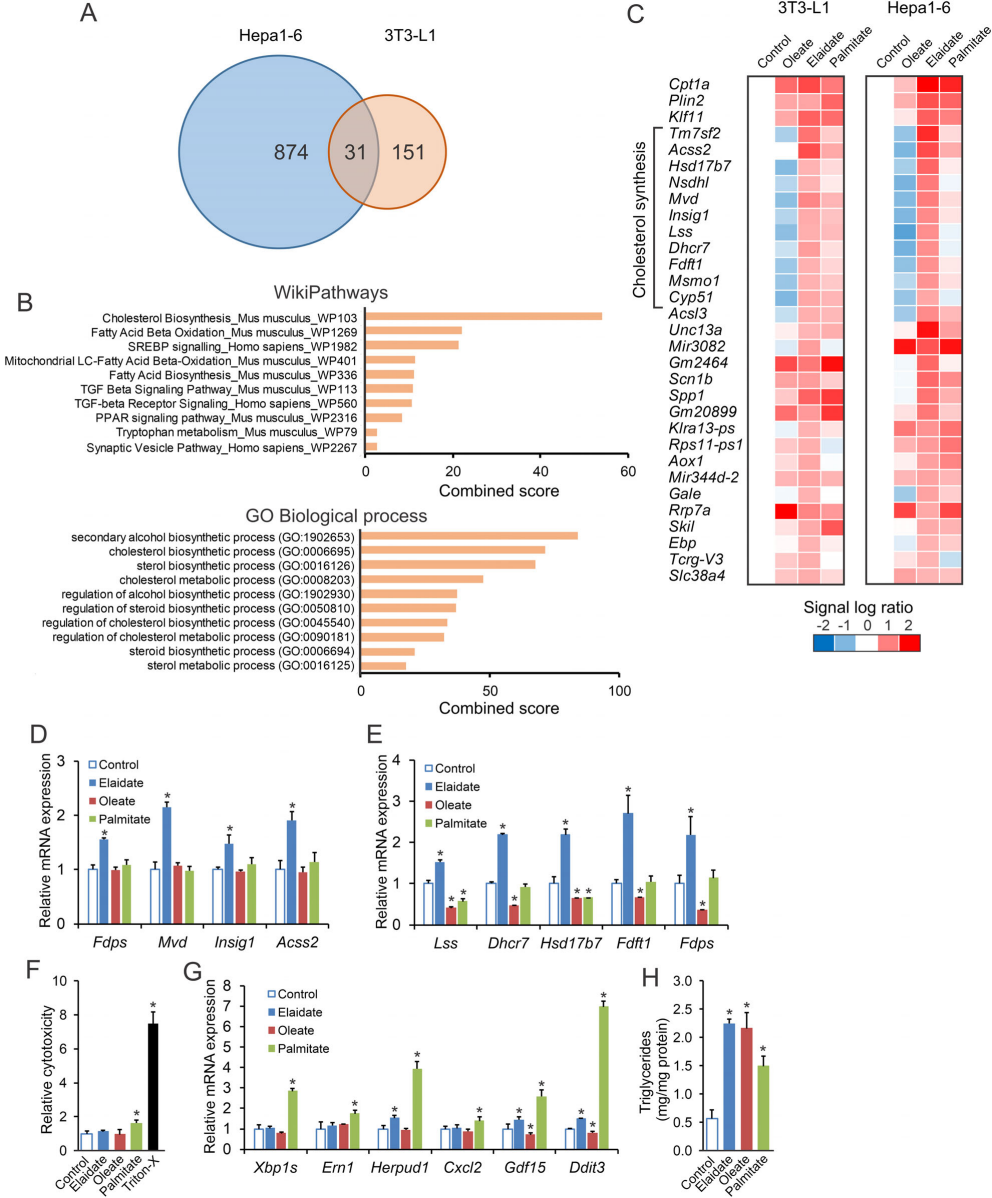
Elaidate induces a cholesterogenic expression profile in differentiated 3T3‐L1 and Hepa1‐6 cells. Differentiated 3T3‐L1 cells were treated with 1 mm fatty acids for 24 h. Hepa1‐6 cells were treated with 500 µm fatty acids for 24 h. A) Venn diagram based on transcriptomics analysis showing the number of genes upregulated by elaidate by at least 1.5‐fold. B) Pathway analysis by Enrichr of the 31 genes commonly upregulated by elaidate in differentiated 3T3‐L1 and Hepa1‐6 cells. C) Heatmap showing relative expression profile of the 31 genes in differentiated 3T3‐L1 and Hepa1‐6 cells after treatment with elaidate, oleate, or palmitate. mRNA expression of cholesterol synthesis genes in D) 3T3‐L1 adipocytes and E) Hepa1‐6 cells. F) Relative cytotoxicity in Hepa1‐6 cells as determined by lactate dehydrogenase assay. G) mRNA expression of ER stress marker genes in Hepa1‐6 cells. mRNA expression was normalized to *36b4*. H) Triglyceride content in Hepa1‐6 cells. Data are mean ± SD; *p < 0.05 relative to control.

To better characterize the molecular pathways activated by industrial trans fatty acids in liver and fat cells, we determined the effects of elaidate on whole genome gene expression in mouse hepatoma Hepa1‐6 cells and mouse 3T3‐L1 adipocytes. For comparison, cells were also treated with oleate and palmitate as representative cis‐unsaturated and saturated fatty acid, respectively. Using a fold‐change cut‐off of >1.5, 31 genes were commonly induced by elaidate in Hepa1‐6 cells and 3T3‐L1 adipocytes (**Figure** 1A). Pathway analysis of the 31 genes by Enrichr showed strong overrepresentation of pathways related to cholesterol synthesis (Figure 1B). Specifically, of the 31 commonly induced genes, 11 genes are involved in the mevalonate and cholesterol synthesis pathway, including *Mvd, Fdft1, Insig1, Dhcr7, Lss* (Figure 1C). In contrast to elaidate, oleate downregulated genes involved in cholesterol synthesis, which was most obvious in the Hepa1‐6 cells, while palmitate showed an intermediate effect (Figure 1C). Overall, oleate was less potent and palmitate was more potent than elaidate in inducing gene expression in Hepa1‐6 cells (Figure S1A, Supporting Information). Few genes were commonly induced in Hepa1‐6 cells and 3T3‐L1 adipocytes by oleate, while many genes were commonly induced in the two cell types by palmitate (Figure S1A, Supporting Information). Among the commonly induced genes by palmitate, there was a marked overrepresentation of pathways related to the unfolded protein response/ER stress (Figure S1B, Supporting Information). QPCR confirmed the induction of cholesterogenic genes by elaidate in 3T3‐L1 adipocytes (Figure 1D) and Hepa1‐6 cells (Figure 1E), which was further verified in undifferentiated and differentiating 3T3‐L1 cells (Figure S2, Supporting Information). Since the gene expression changes were most robust in the Hepa1‐6 cells, we used Hepa1‐6 cells for further investigation into the cellular mode of action of elaidate. Of note, elaidate caused minimal cytotoxicity (Figure 1F) and minimally induced ER stress markers (Figure 1G) in comparison with palmitate, confirming previous data in RAW264.7 macrophages.^20^ Interestingly, elaidate was equally potent as oleate in promoting lipid accumulation in Hepa1‐6 cells, while palmitate was less potent (Figure 1H).

### 3.2 Cholesterogenic Effect of Elaidate is Dependent on SREBP2 and SCAP

**Figure 2:**
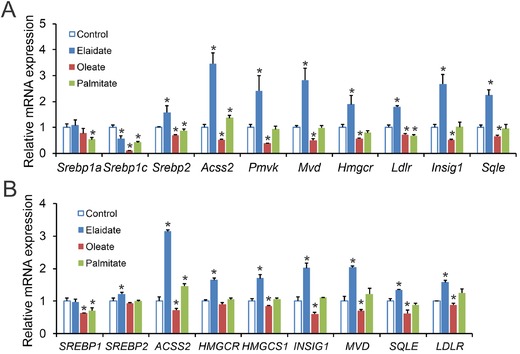
Elaidate induces expression of cholesterogenic genes in murine and human hepatoma cells. A) mRNA expression of cholesterogenic genes in Hepa1‐6 cells treated with elaidate, oleate, or palmitate at 500 µm for 24 h. B) mRNA expression of cholesterogenic genes in Huh7 cells treated with elaidate, oleate, or palmitate at 500 µm for 24 h. mRNA expression was normalized to *36b4*. Data are mean ± SD; *p < 0.05 relative to control.

The expression of cholesterogenic genes is under control of the transcription factors SREBP2 and to a lesser extent SREBP1. Accordingly, we examined the effect of elaidate on the expression of *Srebp1* and *Srebp2*, as well as on their target genes. Compared to control, elaidate significantly upregulated the expression of *Srebp2* and target genes involved in cholesterol synthesis, including *Hmgcr*, *Acss2, Pmvk, Mvd, Fdft1, Ldlr*, and *Sqle* (**Figure** 2A). Elaidate did not induce expression of *Srebp1*. In fact, elaidate downregulated *Srebp1c* (Figure 2A), and had minimal effect on the expression of SREBP1c target genes involved in fatty acid synthesis (Figure S3, Supporting Information). Consistent with the transcriptomics data, oleate downregulated the expression of cholesterogenic genes, while palmitate showed a modest to no effect relative to control (Figure 2A). Similar effects of elaidate on cholesterogenic genes were observed in human Huh7 hepatoma cells (Figure 2B).

**Figure 3:**
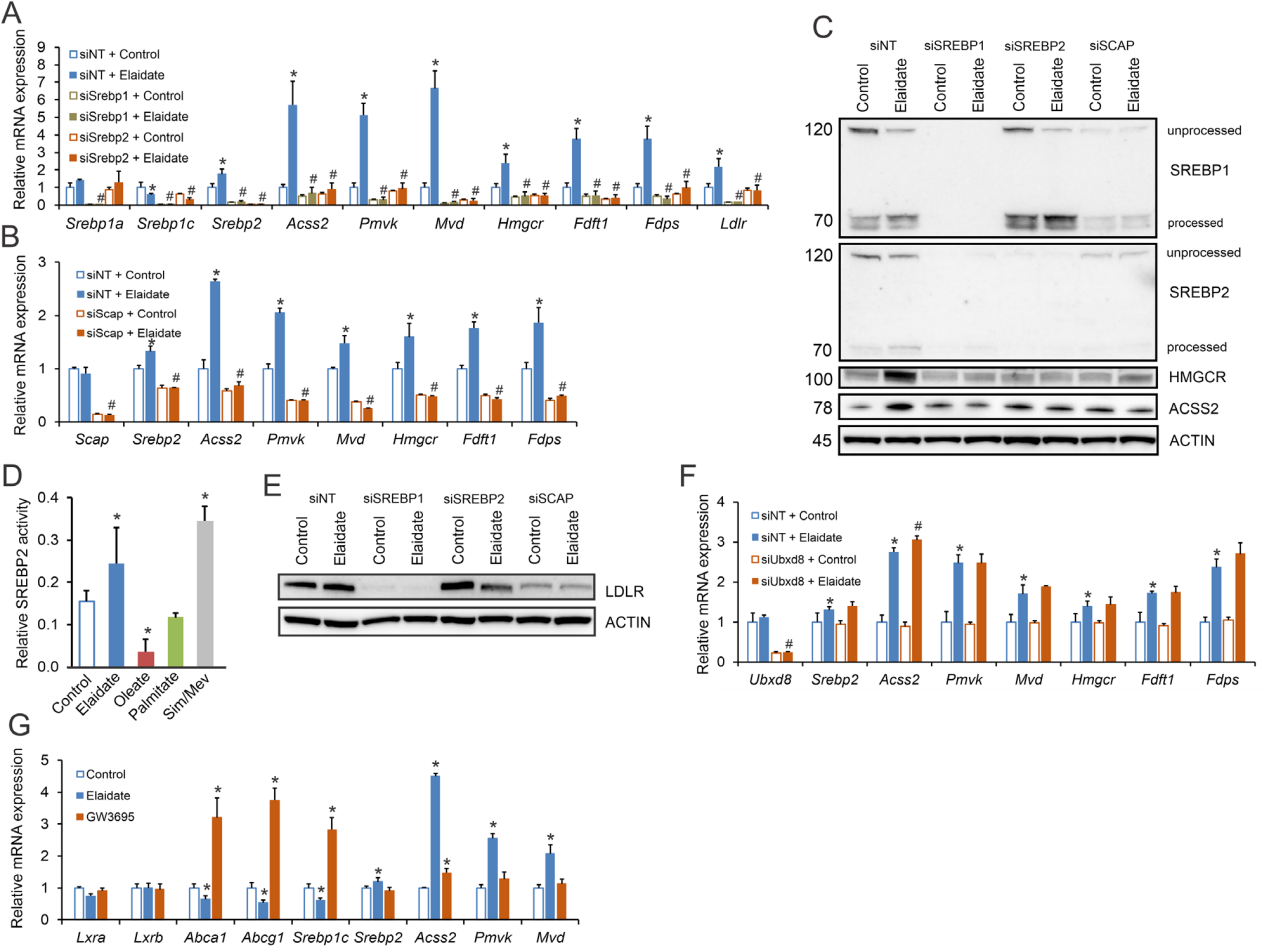
Elaidate induces cholesterogenic gene expression via SREBP2 and SCAP. A) mRNA expression of cholesterogenic genes in Hepa1‐6 cells treated with elaidate and siRNA against *Srebp1* or *Srebp2*. B) mRNA expression of cholesterogenic genes in Hepa1‐6 cells treated with elaidate and siRNA against *Scap*. C) Immunoblot for SREBP1, SREBP2, HMGCR, and ACSS2 in Hepa1‐6 cells treated with elaidate and siRNA against *Srebp1*, *Srebp2*, or *Scap*. β‐Actin served as loading control. D) Relative SREBP2 activity by PLAP‐reporter assay in HEK293 cells. Simvastatin/mevalonic acid (Sim/Mev) at 5 µg mL^−1^ served as a positive control. E) Immunoblot for LDLR in Hepa1‐6 cells treated with elaidate and siRNA against *Srebp1*, *Srebp2*, or *Scap*. β‐Actin served as loading control. F) mRNA expression of cholesterogenic genes in Hepa1‐6 cells treated with elaidate and siRNA against *Ubxd8*. G) mRNA expression of LXR and SREBP target genes in Hepa1‐6 cells treated with elaidate or 1 µm of the LXR agonist GW3695. mRNA expression was normalized to *36b4*. Data are mean ± SD; *p < 0.05 relative to control; #*p* < 0.05 relative to elaidate treatment with non‐targeted siRNA (siNT + Elaidate).

Guided by these results, we probed the role of SREBP1 and SREBP2 in mediating the induction of cholesterogenic genes by elaidate using siRNA. The siRNA‐mediated silencing of *Srebp1* and *Srebp2* completely abrogated the induction of cholesterogenic genes by elaidate (**Figure** 3A), supporting the role of SREBPs. Interestingly, expression of *Srebp2* was not only reduced by siRNA‐mediated silencing of *Srebp2*, but also by silencing of *Srebp1* (Figure 3A). Coupled with the observation that elaidate increased *Srebp2* mRNA but not *Srebp1* mRNA, these data suggest that in these cells the induction of cholesterogenic genes by elaidate is likely mediated by SREBP2.

SREBP‐dependent gene regulation requires SCAP for translocation to the Golgi. SCAP serves as a chaperone protein that facilitates the transport of SREBP to the Golgi apparatus for proteolytic processing and activation. Similar to the effect of *Srebp1/Srebp2* silencing, silencing of *Scap* abrogated the induction of SREBP2 target genes by elaidate, indicating that the effects of elaidate require a functional SCAP–SREBP axis (Figure 3B). The role of the SCAP–SREBP pathway in the regulation of cholesterogenesis by elaidate was further investigated at the protein level. Specifically, elaidate increased the levels of activated SREBP1 and SREBP2, as well as the protein levels of SREBP targets HMGCR and ACSS2 (Figure 3C). Copying the mRNA data, knockdown of either isoforms of *Srebp* or *Scap* abolished the induction of ACSS2 and HMGCR by elaidate. Further in line with the mRNA data, silencing of *Srebp1* resulted in a marked reduction in SREBP2, yet the opposite was not the case (Figure 3C).

To further demonstrate that elaidate enhances the processing and activation of SREBP2, we performed a PLAP‐reporter assay in HEK293 cells, which allows monitoring of translocation and subsequent processing of SREPB2 to its active form.^42^ In this assay, elaidate significantly enhanced the processing of SREBP2 to an extent comparable to that observed with statins (Figure 3D). By contrast, oleate decreased SREBP2 activity. Overall, our data suggest that the stimulatory effect of elaidate on cholesterogenic gene expression is mediated by the SCAP–SREBP2 pathway.

Intriguingly, despite upregulating *Ldlr* mRNA, elaidate did not affect LDLR protein (Figure 3E). Furthermore, elaidate reduced LDLR protein in Hepa1‐6 cells treated with *Srebp2* siRNA but not *Srebp1* or *Scap* siRNA (Figure 3E). These data suggest that, in addition to inducing cholesterogenic gene expression, elaidate has a suppressive effect on LDLR protein levels, which is independent of SREBP2 and dependent on SREBP1 and SCAP.

### 3.3 Cholesterogenic Effect of Elaidate is Independent of UBXD8 and LXR

Having established that elaidate requires an intact SCAP–SREBP axis, we evaluated two potential mechanisms by which this fatty acid may stimulate SREBP signaling. First, we considered the possibility that elaidate requires UBXD8 for its activity, as previous studies have proposed this protein to serve as a sensor for unsaturated fatty acids.^36^ UBXD8 is an ER membrane‐associated protein that promotes SREBP processing by degrading insulin‐induced gene 1 (INSIG1), an inhibitor of SCAP–SREBP translocation. However, as shown in Figure 3F, *Ubxd8* mRNA was not affected by elaidate, nor did silencing of *Ubdxd8* affect the induction of cholesterogenic genes by elaidate, suggesting that the induction is independent of UBXD8 (Figure 3F). Alternatively, we hypothesized that elaidate may inhibit activity of LXR, which regulates *Srebp1c* expression and which has been reported to be inhibited by poly‐unsaturated fatty acids.^49^ To test this idea, we treated Hepa1‐6 cells with the LXR agonist GW3965. LXR activation markedly induced the expression of the established LXR target genes *Abca1, Abcg1*, and *Srebp1c*, whereas elaidate suppressed these genes (Figure 3G). In turn, elaidate strongly induced cholesterogenic genes, whilst GW3965 had minimal to no effect. The above data do not support a role of UBXD8 and LXR in the stimulation of cholesterogenic gene expression by elaidate.

### 3.4 Elaidate Alters Intracellular Cholesterol Metabolism

**Figure 4:**
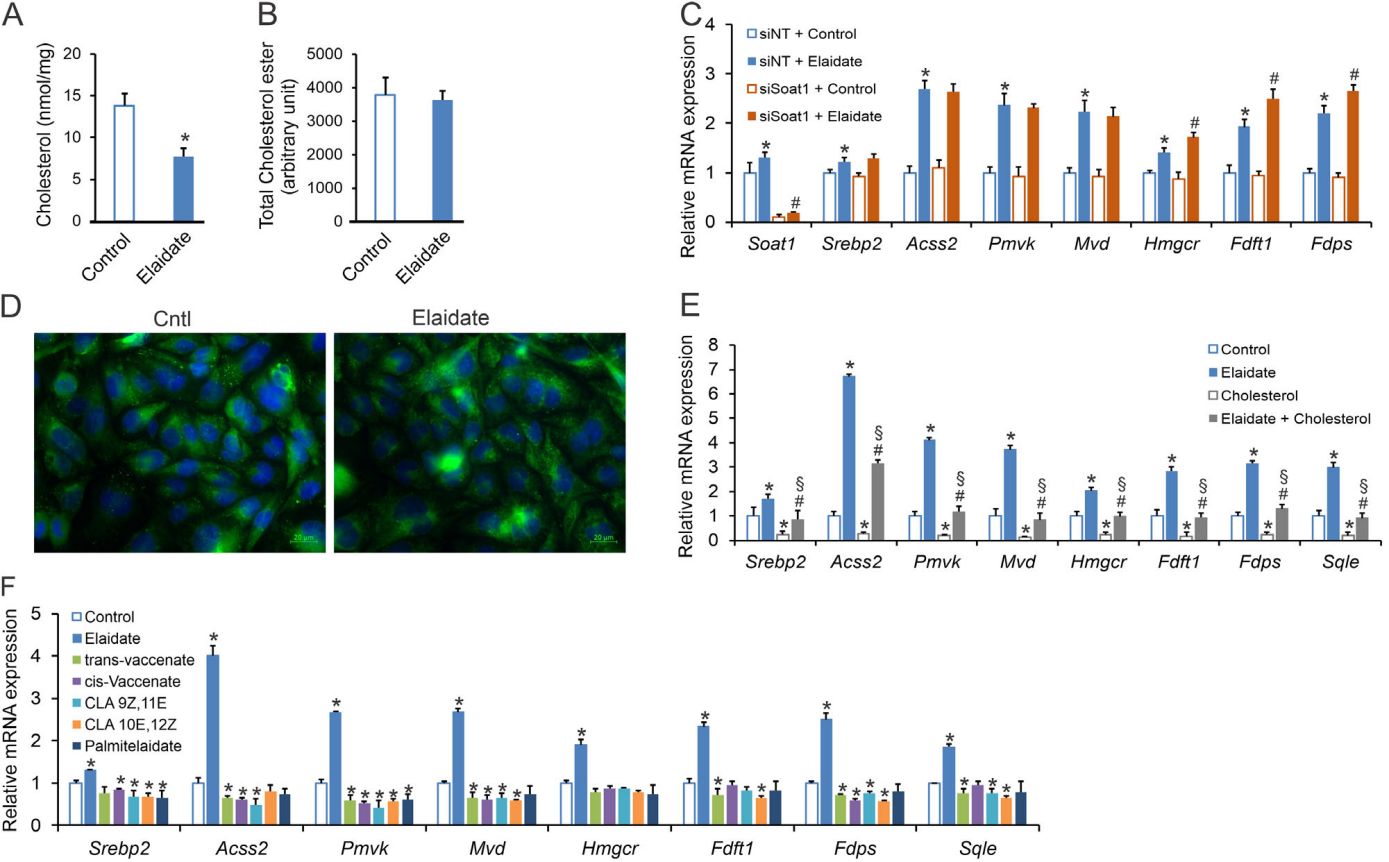
Elaidate alters intracellular cholesterol metabolism in Hepa1‐6 cells. Intracellular levels of A) free cholesterol and B) esterified cholesterol in Hepa1‐6 cells treated with elaidate at 500 µm for 24 h. C) mRNA expression of cholesterogenic genes in Hepa1‐6 cells treated with elaidate and siRNA against *Soat1*. D) SCAP localization in SCAP‐GFP expressing CHO cells treated with elaidate or control. E) mRNA expression of cholesterogenic genes in Hepa1‐6 cells treated for 24 h with 500 µm elaidate and/or 500 µm of exogenous cholesterol. F) mRNA expression of cholesterogenic genes in Hepa1‐6 cells treated with 500 µm of elaidate or other trans fatty acids. mRNA expression was normalized to *36b4*. Images are 200× magnification. Data are mean ± SD; *p < 0.05 relative to control; #*p* < 0.05 relative to elaidate or elaidate treatment with non‐targeted siRNA (siNT + Elaidate); §*p* < 0.05 relative to cholesterol treatment.

To gain further insight into the mechanism behind the cellular effect of elaidate, we determined fluxes and metabolism of intracellular cholesterol. In the ER, cholesterol binds to SCAP to inhibit SREBP transport to the Golgi.^50^, ^51^ A previous study showed that elaidate upregulates sterol O‐acetyltransferase 1 (SOAT1), the enzyme that catalyzes cholesterol esterification.^21^ Accordingly, we hypothesized that elaidate constitutively turns on SREBP signaling by promoting the esterification of cholesterol, thereby decreasing intracellular free cholesterol levels. While elaidate significantly reduced intracellular free cholesterol levels (**Figure** 4A), levels of cholesterol esters remained unaltered (Figure 4B). Furthermore, siRNA‐mediated knockdown of *Soat1* did not abrogate the ability of elaidate to upregulate the expression of SREBP2 target genes (Figure 4C), indicating that the activation of the SCAP–SREBP2 pathway by elaidate is not dependent on cholesterol esterification.

Next, we considered the possibility that elaidate directly influences SCAP translocation. To test this, we studied CHO cells that stably produce SCAP‐GFP.^44^ In these cells, treatment with elaidate did not change the cellular distribution of SCAP (Figure 4D). Alternatively, elaidate may modulate cholesterol sensing by SCAP. To test this notion, we increased intracellular levels of cholesterol by delivering exogenous cholesterol, either in the absence or presence of elaidate. As expected, elaidate induced cholesterogenic gene expression, while exogenous cholesterol had a marked suppressive effect on the same set of genes (Figure 4E). In the presence of elaidate, exogenous cholesterol still significantly reduced cholesterogenic gene expression, suggesting that elaidate does not completely abolish the cholesterol‐sensing ability of SCAP. However, compared to the cholesterol‐only condition, adding elaidate significantly upregulated cholesterogenic gene expression, suggesting that elaidate desensitizes SCAP to cholesterol (Figure 4E). Taken together, these results suggest that elaidate activates the SCAP–SREBP2 pathway through complementary mechanisms that include lowering intracellular free cholesterol levels and desensitizing SCAP to cholesterol.

Finally, we examined whether the stimulatory effect of elaidate on cholesterogenic genes can be generalized to dietary trans fatty acids as a group. Remarkably, elaidate had distinct effects from natural trans fatty acids. Whereas the industrially produced elaidate upregulated cholesterol synthesis genes, natural trans fatty acids such as trans‐vaccenate, conjugated linoleate, and palmitelaidate did not show such an effect (Figure 4F). In fact, if anything, these naturally occurring trans fatty acids mildly decreased expression of these genes. Our data thus indicate that elaidate, but not ruminant trans fatty acids, activate the SCAP–SREBP2 pathway in cultured hepatoma cells.

### 3.5 Trans Fat Feeding Causes Lipoatrophy and Hepatomegaly in Mice

**Figure 5:**
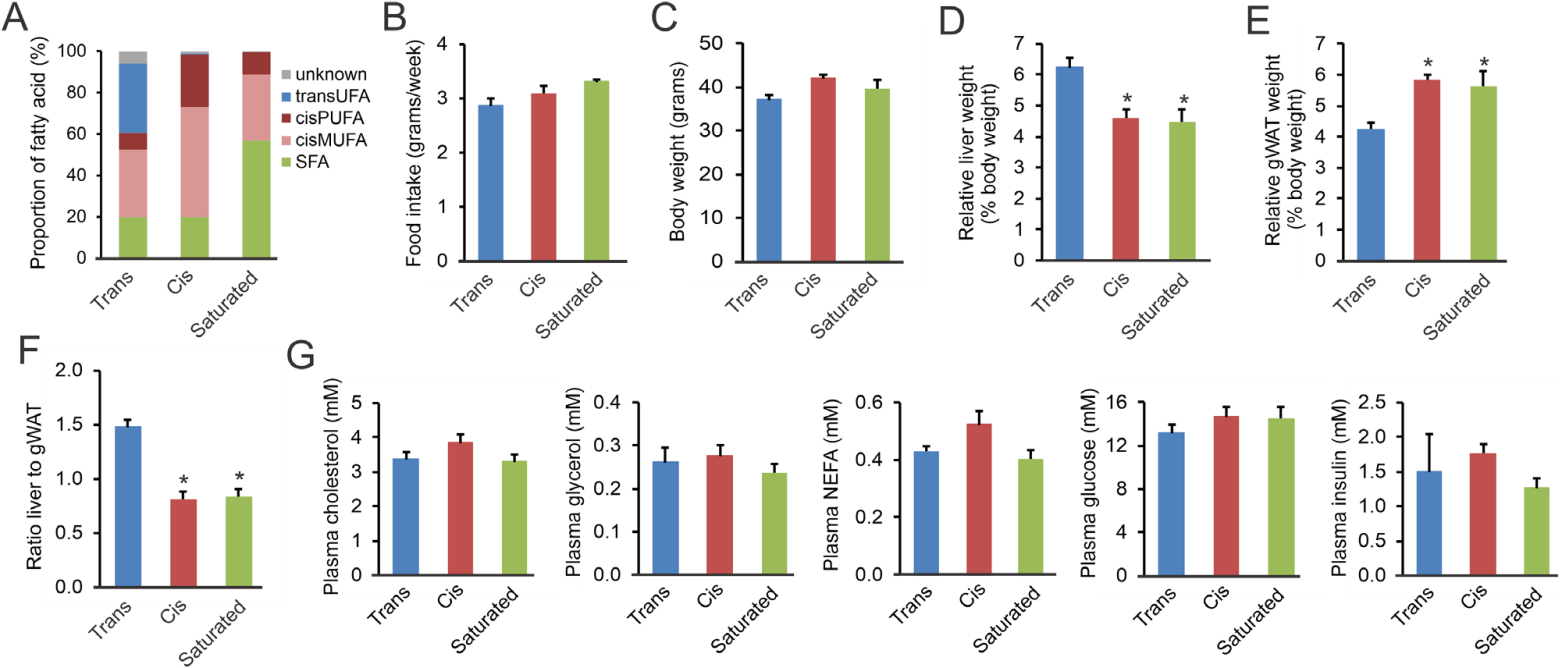
Anthropometric measurements and plasma metabolites in mice fed the different high fat diets for 7 weeks. A) Profile of relative content of different fatty acids in the test diets. TFA, trans‐unsaturated fatty acids; PUFA, poly‐unsaturated fatty acids; cisMUFA, cis mono‐unsaturated fatty acids; SFA, saturated fatty acids. B) Average weekly food intake during the 7‐week diet intervention. C) Final body weights. D) Relative liver weight. E) Relative weight of gonadal white adipose tissue (gWAT). F) Ratio of liver to gWAT weight. G) Plasma levels of cholesterol, glycerol, non‐esterified fatty acids (NEFA), glucose, and insulin. Data are mean ± SEM; *N* = 8 mice per group; *p < 0.05 relative to Trans group.

In a separate set of studies, we aimed to investigate the effects of industrial trans fatty acids on the liver in vivo. To that end, male C57Bl/6 mice were randomly assigned to one of the three calorically equivalent diets enriched in different types of fatty acids: a Trans diet, a Cis diet, or a Saturated diet. The Trans diets consisted of 30–35% trans‐unsaturated fatty acids originating from partially hydrogenated soybean oil (**Figure** 5A and Table S3, Supporting Information). The Cis diet contained equivalent amount of saturated fatty acids as the Trans diet, but with cis mono‐ and poly‐unsaturated fatty acids replacing the trans‐unsaturated fatty acids. The Saturated diet contained about 60% saturated fatty acids, but with equivalent amounts of cis mono‐ and poly‐unsaturated fatty acids as the Trans diet. Overall, about 60–65% of the fatty acid composition was similar between the three diets, with the remaining 35–40% being taken up by either trans‐unsaturated fatty acids, cis‐unsaturated fatty acids, or saturated fatty acids (Figure 5A). Food intake (Figure 5B) and body weight (Figure 5C) were not significantly different between the three groups of mice. In comparison to the mice fed the Saturated and Cis diets, the mice fed the Trans diet had a significantly higher relative liver weight (Figure 5D), but significantly lower relative gonadal fat weight (Figure 5E). Consequently, the ratio of liver to gonadal fat weight was approximately twofold higher in the mice fed the Trans diet (Figure 5F). No significant differences in plasma levels of cholesterol, glycerol, NEFA, glucose, and insulin were observed between the Trans group and the Cis and Saturated groups (Figure 5G).

### 3.6 Trans Fat Feeding Promotes Non‐Alcoholic Fatty Liver Disease

**Figure 6:**
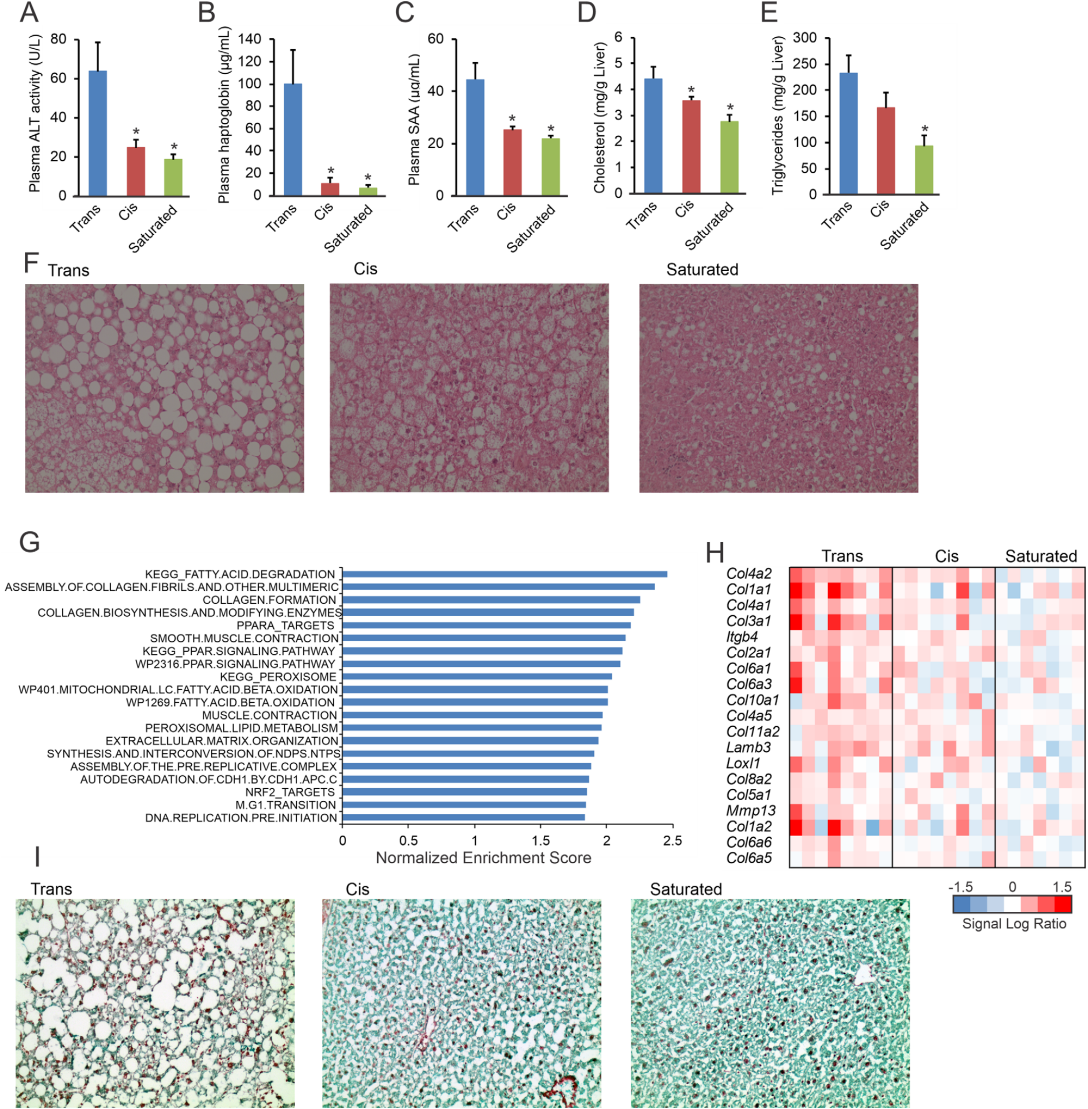
Increased liver damage, steatosis and fibrosis in mice fed the Trans diet. A) Plasma levels of alanine aminotransferase (ALT) activity. Plasma levels of the A) acute phase proteins haptoglobin and B) serum amyloid A. Quantification of hepatic content of D) cholesterol and E) triglycerides. F) Representative images of liver steatosis by hematoxylin and eosin staining. G) Top 20 upregulated gene sets in livers of mice fed the Trans diet, determined by gene set enrichment analysis. Gene sets were ranked according to normalized enrichment score. H) Expression profile of genes within the gene set Assembly of Collagen Fibrils. I) Representative images of collagen staining using Fast Green FCF/Sirius Red. Images are 200× magnification. Data are mean ± SEM; *N* = 8 mice per group; *p < 0.05 relative to Trans group.

**Figure 7:**
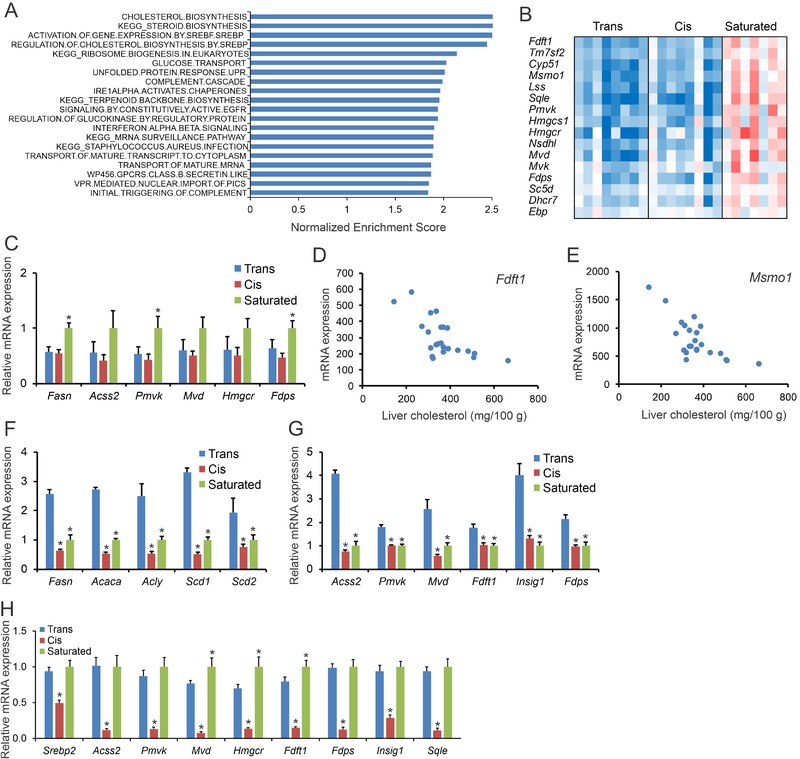
Reduced cholesterogenic gene expression in livers of mice fed the Trans diet. A) Top 20 downregulated gene sets in livers of mice fed the Trans diet for 7 weeks, determined by gene set enrichment analysis. Gene sets were ranked according to normalized enrichment score. B) Expression profile of genes within the gene set Cholesterol Biosynthesis. C) Relative mRNA expression by qPCR of cholesterol synthesis genes in the liver of mice fed the three different types of diets. Strong negative correlation between hepatic cholesterol levels and liver expression of the cholesterol synthesis genes D) *Fdft1* and E) *Msmo1* in the mice fed the three diet. Relative mRNA expression of F) fatty acid synthesis genes and G) cholesterol synthesis genes in the gonadal adipose tissue of mice fed the three different types of diets. H) mRNA expression of cholesterogenic genes in liver of mice fed overnight with the Trans, Cis, or Saturated diets. mRNA expression was normalized to *36b4*. Data are mean ± SEM. *N* = 8 mice per group for the chronic feeding study and *N* = 10 mice per group for the overnight feeding study. *p < 0.05 relative to Trans group.

Remarkably, plasma ALT activity was significantly higher in the Trans group than in the Cis and Saturated groups, suggesting enhanced liver damage (**Figure** 6A). In addition, plasma levels of the acute phase proteins haptoglobin (Figure 6B) and SAA (Figure 6C) were markedly higher in the mice fed the Trans diet. Furthermore, hepatic triglycerides and cholesterol levels were higher in the Trans group compared to the Saturated group (Figure 6D,E). Supporting the quantitative triglyceride analysis, H&E staining revealed enhanced steatosis in the livers of mice fed the Trans diet in comparison with mice fed the Saturated and Cis diets (Figure 6F). To further characterize the hepatic phenotype in the three groups, we performed transcriptomics analysis. Gene set enrichment analysis showed elevated expression of collagen formation pathways and fatty acid degradation pathways in the mice fed the Trans diet (Figure 6G). The higher expression of collagen‐related genes in the Trans group compared to the Cis and Saturated groups is visualized in Figure 6H. Consistent with the transcriptomic results, hepatic collagen staining was most pronounced in the mice fed the Trans diet (Figure 6I), supportive of a more marked NAFLD phenotype. Interestingly, gene set enrichment analysis showed decreased expression of the cholesterol synthesis pathway in the mice fed the Trans and Cis diets compared to the Saturated diet (**Figure** 7A,B), which was confirmed by qPCR (Figure 7C). The expression of cholesterol synthesis genes was highly negatively correlated with hepatic cholesterol levels, suggesting that cholesterogenic gene expression is mainly determined by hepatic cholesterol levels (Figure 7D,E). In contrast to liver, in gonadal adipose tissue the expression of genes involved in lipogenesis (Figure 7F) and cholesterogenesis (Figure 7G) was markedly higher in the Trans group than in the Cis and Saturated groups. This lipo‐ and cholesterogenic effect of the Trans diet was recapitulated in the inguinal adipose tissue depot (Figure S4, Supporting Information). Taken together, these data show that the Trans diet led to more advanced NALFD as compared to the Cis and Saturated diets, as shown by elevated ALT activity, elevated liver triglyceride and cholesterol levels, and increased expression of fibrosis‐related genes.

Since lower cholesterogenic gene expression in the liver after chronic high fat feeding seemed to be mainly driven by higher hepatic cholesterol levels, we determined the acute effect of the different diets on hepatic gene expression. To this end, after a 6 h fast, we fed mice overnight with the Trans diet, the Cis diet, or the Saturated diet. While cholesterogenic gene expression was similar between the Trans and the Saturated group, the expression of cholesterogenic genes was markedly lower in mice fed the Cis diet (Figure 7H). These data suggest that in the short term, industrial trans‐unsaturated and saturated fatty acids stimulate the SREBP pathway in mouse liver in comparison with cis‐unsaturated fatty acids.

## 4 Discussion

The major outcomes of our studies are: 1) a diet enriched in industrial trans fatty acids promotes NAFLD. Specifically, we find that feeding mice a diet enriched in industrial trans fatty acids enhances liver steatosis and fibrosis, and raises hepatic triglyceride levels, hepatic cholesterol levels, and plasma ALT activity in comparison with diets enriched in cis‐unsaturated or saturated fatty acids. 2) in vitro, the industrial trans fatty acid elaidate, but not ruminant trans fatty acids, stimulates the cholesterol synthesis pathway in liver cells via activation of the SCAP–SREBP2 axis, presumably by lowering intracellular free cholesterol and desensitizing SCAP to cholesterol.

Using an integrated transcriptomics, lipidomics, and proteomics approach in HepG2 cells, it was previously shown that elaidate induces the expression of several genes involved in fatty acid and cholesterol synthesis.^21^ Here, using siRNA‐mediated gene silencing, we show that the cholesterogenic effect of elaidate is dependent on functional SREBP2 and SCAP. Although silencing of *Srebp1* abolished the induction of cholesterogenic genes by elaidate, the role of SREBP1 is probably indirect by serving as an important transcriptional regulator of SREBP2. Indeed, SREBP2 protein and mRNA were nearly equally silenced by siRNA targeting *Srebp2* and *Srebp1*. Accordingly, the abrogation of the elaidate effect by *Srebp1* silencing is likely due to the concomitant downregulation of SREBP2. This result is in line with studies that show that both SREBP1 and SREBP2 contain a sterol response element (SRE) in their respective promoter regions, which mediates upregulation of the two isoforms in a feed‐forward manner.^52^, ^53^ Also, the reduction in protein levels of SREBP1 and SREBP2 after SCAP knockdown agrees with a previous study reporting profound reductions in mRNA and protein levels of SREBP1 and SREBP2 in mice with liver‐specific deletion of SCAP, due in part to the abrogation of the feed‐forward mechanism that enables SREBPs to regulate their own expression.^54^

It is well established that the SCAP–SREBP pathway is controlled by fatty acids. Evidence indicates that (poly)‐unsaturated fatty acids inhibit SREBP1‐induced lipogenesis in the liver by suppressing *Srebp1* mRNA levels and inhibiting proteolytic processing of SREBP1. The direct target of (poly)‐unsaturated fatty acids was proposed to be UBXD8, an ER membrane–bound protein that facilitates the degradation of INSIG1, which normally sequesters the SCAP–SREBP complex in the ER and prevents its activation.^36^, ^37^ Our data indicate that activation of the SCAP–SREBP2 pathway by elaidate is independent of UBXD8. Our data also indicate that the effect of elaidate is distinct from that of oleate. Whereas oleate suppressed the expression of cholesterogenic genes in Hepa1‐6 and 3T3‐L1 cells, elaidate had the opposite effect. Apparently, the configuration of the double bond in 9‐octadecenoic acid dramatically alters its signaling properties in liver and other cells. The effect of palmitate was intermediate between that of oleate and elaidate in Hepa1‐6 cells, and differed from elaidate in 3T3‐L1 cells, supporting our previous report in macrophages that elaidate and palmitate have distinct signaling properties.^20^

As already indicated, elevated levels of intracellular free cholesterol serve as a negative feedback regulator of SREBP signaling. When intracellular cholesterol levels drop, the interaction between SCAP–SREBP2 and INSIG1 is released, triggering the movement of SCAP–SREBP2 to the Golgi, where SREBP becomes activated.^55^, ^56^ We found that elaidate significantly reduced intracellular free cholesterol levels, likely accounting for the activation of the SCAP–SREBP2 pathway. One possible explanation for the lower free cholesterol levels is enhanced cholesterol esterification. Although *Soat1* has been reported to be induced by elaidate,^21^ silencing of *Soat1* did not abrogate the cholesterogenic effects of elaidate, nor did elaidate increase intracellular levels of cholesterol esters, ruling out enhanced cholesterol esterification as key underlying mechanism. Besides lowering intracellular free cholesterol, elaidate partially but significantly repressed the strong anticholesterogenic effect of exogenous cholesterol, suggesting that elaidate decreases the sensitivity of SCAP to cholesterol. It is possible that this effect is mediated by elaidate‐induced changes in ER membrane composition, for instance involving an altered ratio of phospholipids to cholesterol or changes in fatty acid composition. Consistent with this line of reasoning, it was recently shown that increased incorporation of poly‐unsaturated fatty acids into phospholipids in the ER accelerated SREBP1c processing through a mechanism that required an intact SCAP pathway.^57^ Overall, our data suggest that elaidate activates SREBP2 signaling through complementary mechanisms that include lowering intracellular free cholesterol levels and desensitizing SCAP to cholesterol, possibly involving changes in ER membrane composition.

A number of studies have reported that ruminant trans fatty acids such as trans‐vaccenic acid can activate peroxisome proliferator‐activated receptors (PPARs), a group of master transcriptional regulators of lipid metabolism.^58^, ^59^, ^60^ Our data shows that in contrast to elaidate, natural trans fatty including trans‐vaccenic acid do not activate SREBP signaling, thereby highlighting a molecular distinction between natural and industrial trans fatty acids.

Previously, Koppe and colleagues showed that compared to diets high in saturated fatty acids, a diet high in industrial trans fatty acids markedly increased plasma ALT activity in AKR/J mice, suggesting enhanced liver damage.^61^ Furthermore, studies in different mouse strains revealed that diets rich in industrial trans fatty acids promote hepatic steatosis and fibrosis.^17^, ^62^, ^63^ Our in vivo study, which provides the most detailed characterization of the effects of industrial trans fatty acids on the liver, extends these findings by showing that a diet enriched in trans fatty acids enhances liver steatosis and fibrosis, and raises hepatic triglyceride levels, hepatic cholesterol levels, plasma acute phase proteins, and plasma ALT activity. Accordingly, we now have convincing evidence that industrial trans fatty acids promote NALFD in mice. Based on these observations, feeding a diet rich in industrial trans fatty acids could be considered as a pre‐clinical model for NAFLD.

Our study is also consistent with other studies showing that the trans fatty acid–induced increase in liver mass and hepatic lipid accumulation is accompanied by a decrease in fat tissue mass.^15^, ^64^, ^65^ One potential explanation for the pronounced increase in the liver to fat ratio is that trans fatty acids may be preferentially directed toward the liver at the expense of fat tissue. It can be hypothesized that lipoproteins containing triglycerides enriched in trans fatty acids may be less efficiently hydrolyzed by lipoprotein lipase in fat tissue. As a result, a larger portion of the dietary fat is taken to the liver. Unfortunately, there are no tools to experimentally validate this hypothesis, given the lack of radiolabelled trans fatty acids and triglycerides. An alternative explanation is that differences in liver to fat ratio are related to differences in adipose tissue lipolysis. However, plasma levels of NEFA and glycerol were not significantly different between the groups, suggesting that adipose tissue lipolysis is not different between the groups. Another explanation for the fatty liver is that trans fatty acids promote lipogenesis or inhibit hepatic VLDL‐triglyceride secretion. However, no indications to that effect were observed. Accordingly, the exact cause for the steatosis and the shift of fat from adipose tissue to liver remains unclear.

Our studies also do not allow us to draw firm conclusions about whether trans fatty acids themselves are responsible for the enhanced NASH features or whether the effects could be mediated by the increase in hepatic cholesterol. Indeed, emerging evidence suggests that hepatic free cholesterol is a major lipotoxic molecule that plays a critical role in the development of NASH.^66^, ^67^ Taking into account the damaging role of cholesterol, it is not unreasonable to suggest that an increase in cholesterogenesis may underlie the stimulation of NASH by industrial trans fatty acids.

Given the major differences in experimental set‐up and complexity of the treatments between the in vitro and in vivo studies, one should be careful to directly compare the outcomes of the two experimental approaches. Nevertheless, cis‐unsaturated fatty acids seemed to behave similarly in vivo and in vitro, as the Cis diet and oleate led to lower cholesterogenic gene expression compared to the Saturated diet and palmitate, respectively. By contrast, whereas elaidate induced cholesterogenic gene expression in Hepa1‐6 cells in comparison with palmitate, chronic feeding of the Trans diet reduced cholesterogenic gene expression in liver in comparison with the Saturated diet, while overnight feeding of the Trans diet led to similar cholesterogenic gene expression as the Saturated diet. Compared with oleate, elaidate‐induced cholesterogenic gene expression in vitro, and overnight feeding of the Trans diet led to higher cholesterogenic gene expression than the Cis diet. Intriguingly, chronic feeding of the Trans and the Cis diet led to similar cholesterogenic gene expression. Although caution should be exercised when comparing the outcomes of in vitro and in vivo approaches, it is tempting to attribute the increase in hepatic cholesterol in the Trans group to activation of the SCAP–SREBP pathway. It can be speculated that the Trans diet initially stimulated hepatic cholesterogenesis, leading to the observed elevation in liver cholesterol levels, which in turn caused a compensatory decrease in cholesterogenic gene expression.

Epidemiological studies have found a positive association between intake of trans fatty acids and NAFLD.^12^, ^13^ Our and other animal studies suggest that this association may be causal. Currently, there is no record of a nutritional trial that investigated the effect of industrial trans fatty acids on intrahepatic lipid or markers of liver damage in humans. It is likely that these studies will never be performed due to ethical restrictions.

Industrial trans fatty acids are best known for their ability to raise plasma LDL cholesterol and lower plasma HDL cholesterol levels in human volunteers.^6^, ^68^, ^69^ Due to the very different lipoprotein profiles of mice and humans, effects of specific treatments on plasma lipid levels in mice cannot easily be extrapolated to the human situation. Nevertheless, we anticipated that the molecular mechanism of action of elaidate in liver cells might give some clues about the plasma cholesterol–modulating effect of trans fatty acids. The observed activation of the SCAP–SREBP2 pathway by elaidate is expected to enhance the extraction of LDL from the bloodstream by activating the transcription of *Ldlr*. Indeed, elaidate significantly increased *Ldlr* mRNA in Hepa1‐6 cells, which was mediated by a functional SCAP–SREBP2 pathway. Strikingly, elaidate was found to suppress LDLR protein levels, via a mechanism independent of SREBP2 and dependent on SREBP1. One possibility is that elaidate decreases LDLR protein by enhancing its degradation. Based on these data, it could be hypothesized that SREBP1 drives the expression of a gene that mediates the degradation of LDLR by elaidate. One obvious candidate would be *Pcsk9*. However, mRNA expression levels of *Pcsk9* were not altered by elaidate. Additional studies are necessary to elucidate the mechanism accounting for the suppressive effect of elaidate on LDLR protein. It should be noted that SREBP1 rather than SREBP2 appears to be the primary regulator of LDLR in Hepa1‐6 cells.

In conclusion, we show that industrial trans fatty acids stimulate cholesterogenesis in vitro via activation of the SCAP–SREBP2 pathway. Induction of cholesterogenesis might explain the increase in liver cholesterol and NAFLD in mice fed a diet rich in trans‐unsaturated fatty acids.

## Conflict of Interest

The authors declare no conflict of interest.

## Supporting information

Supplementary material
