# Supplementary material for: Industrial Trans Fatty Acids Stimulate SREBP2‐Mediated Cholesterogenesis and Promote Non‐Alcoholic Fatty Liver Disease
Source: Mol Nutr Food Res. 2019 Aug 7;63(19):1900385. doi: 10.1002/mnfr.201900385 (PMC6790681; doi:10.1002/mnfr.201900385)
Supplement: Supplementary file 1 — Supplementary material [file MNFR-63-na-s001.pdf]

SUPPLEMENTARY MATERIAL

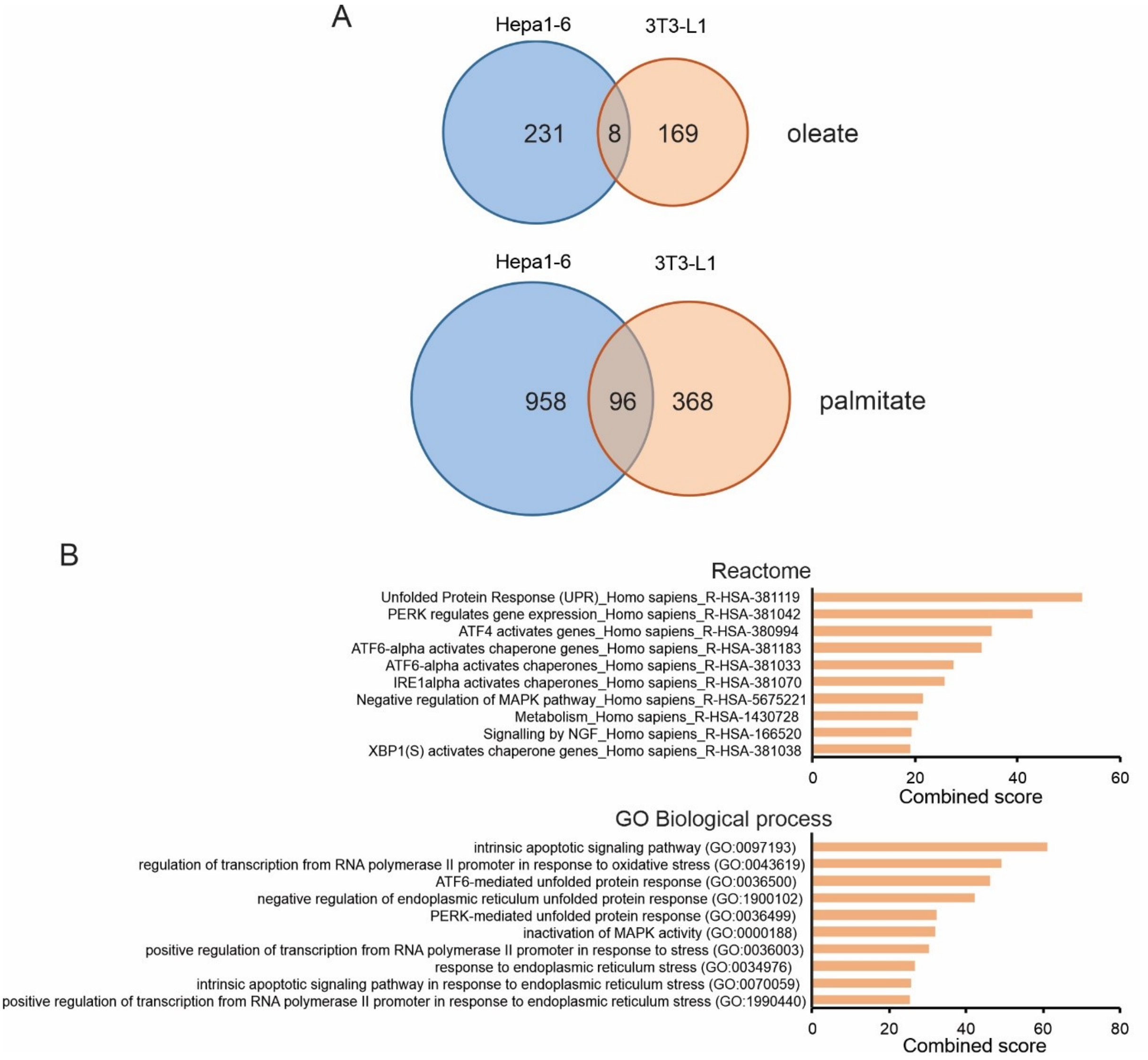

Supplementary Figure 1. Palmitate promotes an ER stress gene expression profile in differentiated 3T3-L1 and Hepa1-6 cells. Differentiated 3T3-L1 cells were treated with 1 mM fatty acids for 24 hours. Hepa1-6 cells were treated with 500 µM fatty acids for 24 hours. (A) Venn diagram based on transcriptomics analysis showing the number of genes upregulated by oleate or palmitate by at least 1.5-fold. (B) Pathway analysis by Enrichr of the 96 genes commonly upregulated by palmitate in differentiated 3T3-L1 and Hepa1-6 cells.

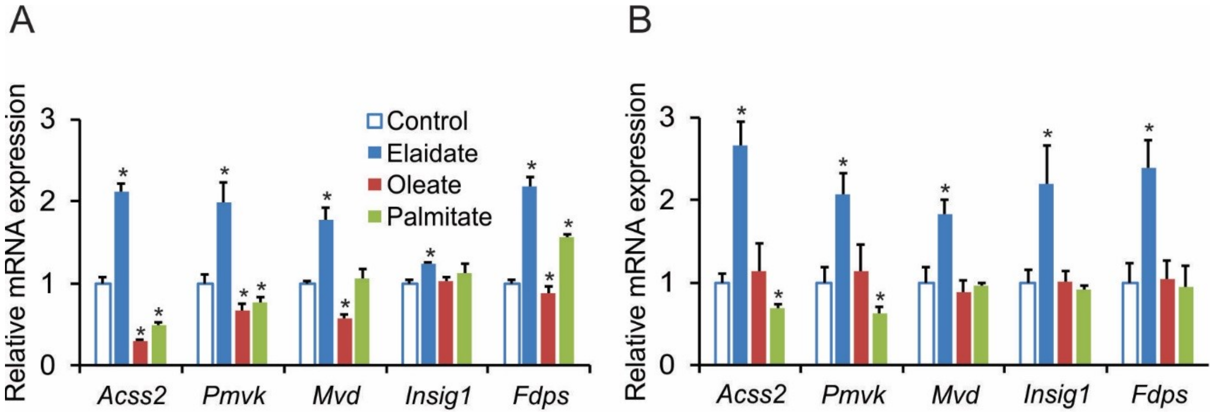

Supplementary Figure 2. Elaidate induces cholesterogenic genes in 3T3-L1 cells. (A) Relative mRNA expression of cholesterol synthesis genes in undifferentiated 3T3-L1 fibroblasts treated with 500 μM of individual fatty acids for 24 hours. (B) Relative mRNA expression of cholesterol synthesis genes in 3T3-L1 cells that were differentiated in the presence of 500 μM of individual fatty acids. mRNA expression was normalized to *36b4*. Data are mean ± SD. *p < 0.05 relative to control.

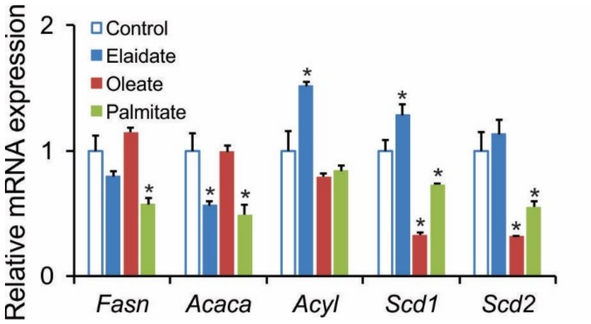

Supplementary Figure 3. Relative mRNA expression of target genes of SREBP1c involved in fatty acid synthesis in Hepa1-6 cells treated with 500 μM of individual fatty acids for 24 hours. mRNA expression was normalized to *36b4*. Data are mean ± SD. *$p < 0.05$ relative to control condition.

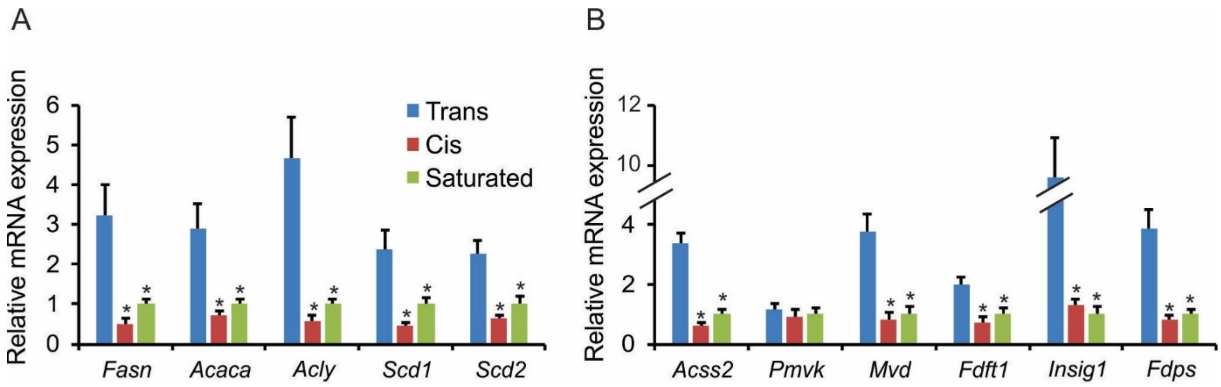

Supplementary Figure 4. Trans diet induces lipogenic and cholesterogenic genes in inguinal adipose tissue. (A-B) Relative mRNA expression of fatty acid synthesis genes (A) and cholesterol synthesis genes (B) in the inguinal adipose tissue of mice fed Trans, Cis and Saturated diets. mRNA expression was normalized to *36b4*. N=8 mice/group. Data are mean ± SEM. *p < 0.05 relative to Trans group.

Supplementary Table 1: Composition of Test Diets

Ingredients (%)	Saturated fat diet	Unsaturated fat diet	Trans fat diet
Cocoa Butter	**23.3060**	0	0
Canola Oil	0	**13.2265**	0
Palm Olein Oil	0	**7.8536**	0
Hydrogenated Soy Oil	0	0	**21.0800**
Casein- Vitamin Tested	23.3060	23.3059	23.3060
Sucrose	19.7090	19.7090	19.7090
Maltodextrin	11.6530	11.6530	11.6530
Dextrin	8.4830	8.4830	8.4830
Powdered Cellulose	5.8270	5.8270	5.8270
Soybean Oil	2.9200	2.9200	2.9200
Potassium Citrate, Tribasic Monohydrate	1.9230	1.9230	1.9230
Calcium Phosphate	1.5150	1.5150	1.5150
AIN-76A Vitamin Mix	1.1650	1.1650	1.1650
DIO Mineral Mix	1.1650	1.1650	1.1650
Calcium Carbonate	0.6410	0.6410	0.6410
L-Cystine	0.3500	0.3500	0.3500
Choline Bitartate	0.2330	0.2330	0.2330
FD&C Yellow 6 Lake (Orange)	0.0300	0.0300	0.0300

Supplementary Table 2: Primer sequences of genes

Genes	Forward primer	Reverse primer
m36b4	ATGGGTACAAGCGCGTCCTG	GCCTTGACCTTTTCAGTAAG
mFasn	GGCATCATTGGGCACTCCTT	GCTGCAAGCACAGCCTCTCT
mAcaca	GCCATTGGTATTGGGGCTTACC	CCCGACCAAGGACTTTGTTG
mAcyl	CCAGTTAATCAAACGTCGAGGA	CTTTGGCCTTGCCGACAGT
mScd1	TAGCCTGTAAAAGATTTCTGCAAACC	CCGGAGACCCTTAGATCGA
mScd2	TTCTCCCGAGAGCTAATGTTCT	TTCTTGCGATACGCCGTGG
mAcss2	AAACACGCTCAGGGAAAATCA	ACCGTAGATGTATCCCCCAGG
mPmvk	AAAATCCGGGAAGGACTTCGT	AGAGCACAGATGTTACCTCCA
mMvd	ATGGCCTCAGAAAAGCCTCAG	TGGTCGTTTTTAGCTGGTCCT
mFdft1	TCCCACTGCTGTGTAACTTCC	TGTCTACAAATTCTGCCATCCC
mInsig1	TGTCGGTTTACTGTATCCCTGT	GGCAAAATCTAATTTGGCACTGG
mFdps	GGAGGTCCTAGAGTACAATGCC	AAGCCTGGAGCAGTTCTACAC
mXbp1s	GAGTCCGCAGCAGGTG	GTGTCAGAGTCCATGGGA
mErn1	ACACCGACCACCGTATCTCA	CTCAGGATAATGGTAGCCATGTC
mHerpud1	CCTGGCTTCTCTGGCTACAC	GTCGGGACAAAAGTTCCTGA
mCxcl2	CCAACCACCAGGCTACAGG	GCGTCACACTCAAGCTCTG
mGdf15	CTGGCAATGCCTGAACAACG	GGTCGGGACTTGGTTCTGAG
mDdit3	CTGGAAGCCTGGTATGAGGAT	CAGGGTCAAGAGTAGTGAAGGT
mSrebp1c	GGAGCCATGGATTGCACATT	CCTGTCTCACCCCCAGCATA
mSrebp2	CTGCAGCCTCAAGTGCAAAG	CAGTGTGCCATTGGCTGTCT
mHmgcr	AGCTTGCCCGAATTGTATGTG	TCTGTTGTGAACCATGTGACTTC
mLdlr	GCATCAGCTTGGACAAGGTGT	GGGAACAGCCACCATTGTTG
mScap	TGGAGCTTTTGAGACTCAGGA	TCGATTAAGCAGGTGAGGTCG
mUbxd8	GAGCAGGATCTAACTCAGGAGC	CAGCAGCCTCCATGTTCCAG
mLxra	CTCAATGCCTGATGTTTCTCCT	TCCAACCCTATCCCTAAAGCAA
mLxrb	CGTGGTCATCTTAGAGCCAGA	GCTGAGCACGTTGTAGTGGAA
mAbca1	AAAACCGCAGACATCCTTCAG	CATACCGAAACTCGTTCACCC

Supplementary Table 2 continued: Primer sequences of genes

Genes	Forward primer	Reverse primer
mAbcg1	CTTTCCTACTCTGTACCCGAGG	CGGGGCATTCCATTGATAAGG
mSoat1	GAAGGCTCACTCATTTGTCAGA	GTCTCGGTAAATAAGTGTAGGCG
mSqle	ATAAGAAATGCGGGGATGTCAC	ATATCCGAGAAGGCAGCGAAC
hGAPDH	GAAGGTGAAGGTCGGAGTC	GAAGATGGTGATGGGATTTC
hSREBP1	ACTTCCCTGGCCTATTTGACC	GGCATGGACGGGTAAATCTT
hSREBP2	AACGGTCATTCACCCAGGTC	GGCTGAAGAATAGGAGTTGCC
hACSS2	CAAGTGTGTCAGTTCAGCAATG	CCACAAGCTCTGGGATCATAGG
hHMGCR	GGACCCCTTTGCTTAGATGAAA	CCACCAAGACCTATTGCTCTG
hHMGCS1	CTCTTGGGATGGACGGTATGC	GCTCCAACTCCACCTGTAGG
hMVD	GGACCGGATTTGGCTGAATG	CCCATCCCGTGAGTTCCTC
hINSIG1	CCTGGCATCATCGCCTGTT	AGAGTGACATTCCTCTGGATCTG
hSQLE	TGACAATTCTCATCTGAGGTCCA	CAGGGATACCCTTTAGCAGTTTT
hLDLR	GACGTGGCGTGAACATCTG	CTGGCAGGCAATGCTTTGG

Supplementary Table 3: Detailed fatty acid profile of diets

Fatty acids	Saturated	Cis-Unsat	Trans-Unsat
C 14:0	0.18	0.44	0.14
C 14:1 9t	0	0	0
C 14:1 9c	0	0	0
C 16:0	23.76	16.16	11.01
C 16:1 9t	0	0	0
C 16:1 9c	0.18	0.26	0
C 17:0	0.28	0	0.17
C 17:1 10c	0	0	0
C 18:0	31.59	2.99	8.23
C 18:1 7+8t	0.11	0	4.28
C 18:1 9t	0	0.11	3.33
C 18:1 11t	0	0	6.66
C 18:1 5c+7c+12t	0	0	5.03
C 18:1 9c+13t	30.98	49.24	23.84
C 18:1 11c+15t	0.87	2.54	2.05
C 18:1 12c	0	0.23	6.85
C 18:1 13c	0	0.10	0.91
C 18:1 15c	0	0	0.65
C 18:2 tt	0	0	0.78
C 18:2 ct	0	0.19	0
C 18:2 tc	0	0.11	0
C 18:2 cc	9.67	19.96	7.56
C 18:3 ttt	0	0	0
C 20:0	1.10	0.55	0.31
C 18:3 tct	0	0	0
C 18:3 ctt	0	0	0
C 18:3 ctc	0	0.09	0
C 18:3 tcc	0	0.19	0
C 18:3 ccc	1.16	5.03	1.00
C 20:1 11c	0	0.84	0.21
Unspecified unsaturated cis/trans	0	0	11.01
Other unknown fatty acids	0.14	1.04	6.03
